# Evaluation of the validity of the physical exercise peer support questionnaire for college students

**DOI:** 10.3389/fpubh.2022.871306

**Published:** 2022-08-01

**Authors:** Lin Luo, Xiuxiong Yang, Xiaojin Zeng, Naiqing Song, Ling Zhou, Liping Zhang, Yongbin Yang, Jie Yang

**Affiliations:** ^1^College of Physical Education, Guizhou Normal University, Guiyang, China; ^2^Basic Education Research Center, Southwest University, Chongqing, China; ^3^East China Normal University-Xuhui Postdoctoral Workstation, Shanghai, China; ^4^College of Exercise and Health, Shandong Institute of Physical Education, Rizhao, China

**Keywords:** college students, physical exercise, peer support, validity, PEPSQ

## Abstract

Peer support for physical exercise is defined as behaviors such as mutual or one-way provision of material help and/or emotional care and companionship between peers in the physical environment and/or physical behavior. The assessment of peer support is complex and based on reasoning. Trustworthy assessment processes need to provide sufficient evidence of validity. The purpose of this study was to organize, collect, and use Kane's validity framework to provide validity evidence for the identification of peer support for physical exercise among college students. The article describes the experience of using the framework in this study, considers data related to the four inferences (scoring, generalization, extrapolation, and implication) that emerge from the assessment process. The findings of the study are then interpreted through the four inferences to determine whether this evidence supports the purpose of this study. Based on Kane's framework to explain the validity process of this study, the study concludes that the evidence in terms of scoring, generalization, extrapolation, and implication supports the use of the PEPSQ for the identification of physical exercise peer support among college students.

## Introduction

The university stage is an important period of individual transition and development, and an important stage of health reserve in adulthood. College students are the future force of national construction, and the physical health of college students has far-reaching significance for national quality improvement and population structure optimization. With the continuous expansion of the enrollment scale of Chinese higher education institutions, the number of college students continues to increase, but the physical health level of Chinese college students shows a gradual downward trend (1, 2). The Report on the Development of Youth Sports in China (2015) points out that the performance of Chinese college students in various physical fitness tests is still at a low level, and the test results of certain items are sometimes inferior to those of secondary school students (3). In 2020, the Chinese Ministry of Education conducted physical fitness review tests on 1.15 million school students. The results of the test showed that about 30% of college students failed the physical fitness test, the highest percentage among all academic levels (4). Scholars point out that effective health education guidance is needed to improve the physical health level of college students, and the physical health level of college students can be improved by encouraging them to actively participate in physical exercise (5). Although the physical and mental health effects of physical exercise have been widely recognized by the public, the lack of participation in physical exercise among college students is still a common phenomenon 2015 physical exercise survey data of college students in 23 countries around the world showed that the proportion of insufficient physical exercise among college students was as high as 41.4%. Among them, the percentage of insufficient physical exercise among Chinese college students was 37.0% (6). Therefore, finding the key elements that potentially affect college students' participation in physical exercise is an important part of developing health education interventions that effectively promote college students' physical exercise participation and improve their physical health.

Peer support belongs to the category of social support. Mead et al. defined peer support as a system of giving and receiving help based on the key principles of respect, shared responsibility, and mutual help (7). Wentzel et al. defined peer support as the mutual or one-way provision of material help and/or emotional care and companionship, among other behaviors (8). A number of studies have found a positive relationship between peer support and individual physical exercise behaviors. For example, Fitzgerald et al. found that the perceived level of peer support played an important role in adolescent physical exercise behavior among adolescents aged 10–18 years (9). Chen et al. found that peer support enhanced self-efficacy and thus promoted physical exercise frequency among students in grades 9–12 (10). Reimers et al. found that peer support levels were associated with frequency of multiple physical exercise behaviors (outdoor play, sports, or walking transportation) among children aged 6–17 years (11). Sylvia-Bobiak et al. found gender differences in the relationship between peer support and physical exercise behaviors among college students. Peer support influenced physical exercise participation more significantly in male college students than in female college students (12). Therefore, understanding an individual's perceived level of peer support may be helpful in promoting individual physical exercise behaviors.

Existing research has developed a number of measurement instruments to identify individuals' perceived peer support. For example, Zimet et al. designed a measure of peer support in their development of the Multidimensional Scale of Perceived Social Support (MSPSS) (13). Mostafaei et al. designed a peer support scale containing five dimensions: informational support, emotional support, instrumental support, feedback, and companionship support (14). Some social support scales are also often used to measure peer support, such as the child and adolescent social support scale (CASSS) (15), the College Student Social Support Scale (16), and the Social Support Rating Scale (17). However, given the complexity of an individual's perceived peer support, conducting accurate and trustworthy assessments can be a challenge. This is because individuals differ in their behaviors such as providing material help and/or emotional care and companionship to each other or singularly in specific contexts or specific behaviors (9). For example, peer support in a health care context often includes emotional, informational, and assessment support. In this setting, emotional support includes expressions of caring, encouragement, careful listening, reflection, reassurance, and often avoids critical or persuasive advice (18). Informational support is the provision of knowledge related to problem solving, including the availability of relevant resources, independent assessment of the problem, alternative courses of action, and guidance on effectiveness (19). Evaluative support, also known as affirmative support, involves the exchange of information related to self-evaluation and includes affirmation of expressions of emotional, cognitive, and behavioral appropriateness (20). Peer support in the workplace, on the other hand, is more concerned with drawing on life experiences, engaging in mutually beneficial discussions, and so on (21). Therefore, it seems essential to conduct context-specific or behavior-specific peer support assessments. To the best of our knowledge of published articles, there are several assessment tools available to identify social support in physical exercise settings. For example, Zhong et al. developed the Exercise Social Support Scale (22), which contains four dimensions, namely emotional support, informational support, instrumental support, and peer support. Sallis et al. developed the Social Support for Exercise Scale, which contains two dimensions: the Family Support for Exercise Scale and the Friend Support for Exercise Scale (23). Farias et al. developed the Social Support for Adolescent physical exercise Scale (ASAFA), which consists of two dimensions: parental support and friend support (24). However, most of the existing assessment tools consider peer support as a dimension of social support and do not provide a more detailed assessment of the emotional, informational, and behavioral support provided by peers.

Given the current physical health status of Chinese college students, there is a need to develop an assessment tool that can effectively identify the perceived level of peer support among college students in a physical exercise setting. Therefore, this study aimed to design a preliminary peer support questionnaire for physical exercise among Chinese college students and to collect validity evidence for the questionnaire based on the Kane framework (25). The validity evidence included four inferential processes of scoring, generalization, inference, and influence, thereby objectifying the subjectivity and qualitative nature of college students' perceived levels of peer support in physical exercise settings.

This study follows the Kane framework to produce a workflow that illustrates how it can be used to conduct a validity validation study of the PEPSQ. In a later section of the article, this study describes the study's evaluation setting and evaluation strategy, defines the study's key variables, specifies the study's hypotheses and the evidence collected to test those hypotheses. The results of the evaluation process are also compared to the initial arguments. This study also reflects on and discusses the gaps in the discussion of this study's application of the framework. Using this study's evaluation process, this study demonstrates how to collect empirical data and report the judgment process for PEPSQ validity.

## Research method

In collecting and evaluating the validity evidence for the PEPSQ, this study applied the Kane validity framework. In accordance with the characteristics of the Kane framework, decisions must be made prior to the study as to which inferences need to be considered and judgments must be made as to whether the evidence obtained is favorable or unfavorable in the absence of clear guidelines. This research team tested, documented, and reflected on the challenges and final decisions in applying the theory to practice. The following is a specific description of the methodology of this study.

### Research overview

**Figure 1:**
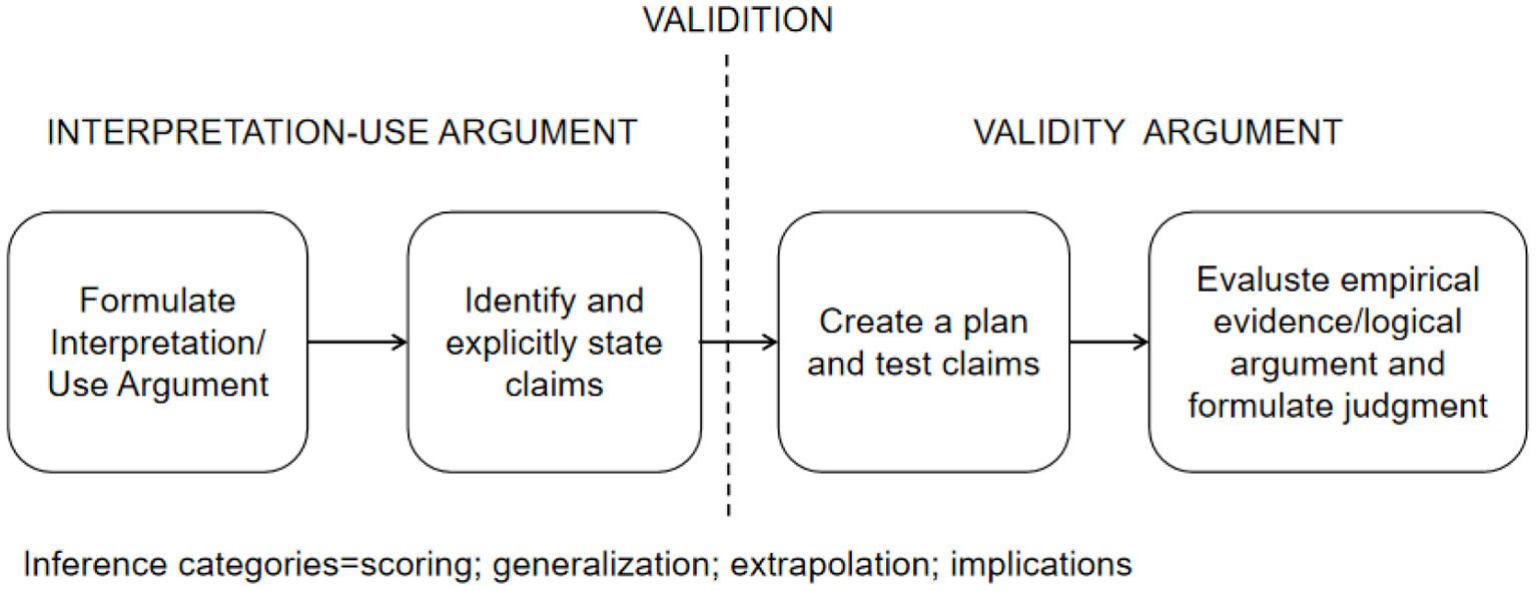
Schematic illustrating the process of validation, including specification of the inherent claims associated with the interpretation-use argument (from left to right, boxes 1 and 2) and evaluation of those claims (boxes 3 and 4).

Figure 1 illustrates the interpretation of the validity process for this study using the Kane framework.This study focused on the level of perceived peer support among university students in a physical exercise environment. A questionnaire containing five measurement dimensions was initially designed for this study to determine the measurement structure of peer support. The questionnaire addressed interest support, material support, emotional support, behavioral support and information support from peers as perceived by university students in the physical exercise environment. The plan of this study was for the assessor to identify the level of peer support of college students in the physical exercise environment through the PEPSQ and to predict college students' physical exercise behavior based on the results of the PEPSQ scores. Based on this interpretation and use, this study illuminates much of the evidence of validity in the process of constructing the PEPSQ. Based on the Kane framework, this study organizes four validity arguments: scoring, generalization, extrapolation and implication.

In essence, this study traced the assessment of perceived peer support in physical exercise settings among college students. From scoring a single observed entry (scoring), to using observed scores to generate an overall test score representing performance in the testing environment (generalization), to making inferences about what the test score might imply about real-life performance (extrapolation), and then prejudging and making decisions about this information (implication). This study presents this process of validity argumentation using Table 1. Scoring examines the relationship between observed performance and the score or rating generated by that performance; generalization examines the link between a sample of observed performance and the broader domain of all possible performance in the assessment setting; extrapolation focuses on the link between assessment results and other measures of similar performance domains; and implication making examines the integrity of the process leading to the decision and the individual, project or societal Consequences (26).

**Table 1:** Specific evidence to support validity arguments.

	**Scoring**	**Generalization**	**Extrapolation**	**Implication**
Definition of Kane (26)	Rule is appropriate Rule is applied as specified Scoring is free of bias Data fit the scaling todel	Sample is representative of universe of possible observations Sample is large enough to control for random error	Observed score is related to the target score No systematic errors likely to undermine the extrapolation	Implications (interpretations) are appropriate Properties of scores support the implications (interpretations) associated with the label
Operational sources of evidence	Development of scoring dimensions/development of selection items Evidence of independence of scoring dimensions Distinguishability of scored items Evidence of scoring reliability Quality control of scoring	Internal consistency reliability across projects Assessment of sources of measurement error Sampling of observations (number of items or sites, breadth of content) Sample size	Relationships with other variables/measures (correlation with other scores) Development of items to reflect the full breadth of real-life tasks Retesting performance	Impact on the physically exercise person (i.e., viewing the act of assessment as an intervention) Impact on the project Accurate classification of individuals Standard setting process

### Analysis plan

This study organized the data collection and analysis of the study by inference categories, scoring, generalization, extrapolation, and implications in the following steps.

In this study, peer support for physical exercise was defined as the behavior of peers providing material help and/or emotional care and companionship in the physical exercise environment and/or physical behavior, either mutually or unidirectionally. Firstly, the core elements of each dimension of the existing questionnaire were analyzed by the subject members based on the literature, for example, the interest support dimension includes interest in the direction of exercise purpose, project hobbies, etc. Secondly, the relevant questions were developed according to the core elements. The sources of questions mainly include the following ways: (1) borrowing and adapting relevant items from established peer support measurement tools at home and abroad, such as the Friendship Quality Scale for Youth Sports in China (SFQA-C) (22), the Questionnaire on Social Support, Motivation and Participation in Sports for Youth (27), and the Questionnaire on the Status of Peer Support and physical exercise for Children and Youth (28), etc. (2) Based on the research and review in the field of factors influencing physical exercise among college students, representative contents were extracted and compiled into test items. (3) Relevant test items were compiled based on the additional contents and expressions of front-line physical education and health course teachers and college students in the open-ended questionnaire. Finally, the topics that best fit the operationalized definition of each dimension and have less crossover between dimensions were selected after discussion by the group, and experts in the field of physical and health education were invited to evaluate the content validity and make suggestions for modification. Finally, the initial questionnaire containing five dimensions and 42 questions was developed.

The first test had a total of three assessors. The second test had a total of six assessors. The assessors were current graduate students. All assessors received training on the item description 1 week prior to the assessment. The training included the conceptual framework of the assessment design, the role of the assessors, and a detailed description of the scoring instrument and how to apply it.

The assessor is primarily responsible for administering the questionnaire. The completion of the assessment questionnaire was done by the subjects themselves. Raters were asked to avoid sharing perceptions of any performance or sharing assigned scores in order to avoid calibration of the rater over time. All data were completed and data collected directly through the electronic questionnaire platform. Each question was scored from 1 (not at all) to 5 (fully). Data analysis calculates the subject's score for individual questions, as well as the score for each dimension and the total score.

The study used the Mack electronic questionnaire platform for data collection. The first survey came from college students in multiple universities in the author's city (352 valid questionnaires). This sample was used to conduct a preliminary exploration of the dimensions of the test questionnaire. The second test came from college students in six Chinese provinces and cities (1,219 valid questionnaires). This sample was used to examine the stability of the questionnaire dimensions and the similarity of students' test scores. The basic information of the respondents of the two surveys is shown in Table 2.

**Table 2:** Personal information of survey respondents.

**Test**	**Gender**	***N***	**Age (M ±SD)**	**Household registration type**	
				**Citie and town**	**Rural**
First test	Male	178	20.12 ± 1.40	16.9%	83.1%
	Female	174	19.94 ± 1.12	20.1%	79.9%
	Total	352	20.03 ± 1.27	18.5%	81.5%
Second test	Male	517	20.15 ± 1.34	16.8%	83.2%
	Female	702	20.02 ± 1.34	20.9%	79.1%
	Total	1,219	20.09 ± 1.34	18.8%	81.2%

This study hoped to identify the perceived level of peer support among college students in physical exercise settings. Therefore, this study sought additional measures to help validate the performance of the PEPSQ in the real world.The Exercise Social Support Scale (22) developed by Zhong et al. was used in a correlation analysis with this questionnaire to test the inferential effects of the results of this questionnaire test.

The purpose of this study is to construct an assessment tool that can effectively identify the level of perceived peer support among college students in physical exercise setting. Therefore, the peer support scores obtained through the PEPSQ should be able to predict the physical exercise behavior of college students in a real environment. At the same time, the measurement results of PEPSQ should have a certain degree of stability. In this study, 48 college students were randomly selected from the second test for retesting, which was used to test the stability of the PEPSQ assessment results.

## Reseach results

Kane's validity framework emphasizes a chain of inferences from score generation to decisions about the ratee, a chain that can be conceptualized as the path that must be followed before sufficient evidence can be obtained. Therefore, the present study reports the results of this study guided by this stepwise conceptualization process.

### Evidence of scoring

In this study, a questionnaire analysis was conducted using test data from 352 college students to determine the dimensions and items of the PEPSQ. The independence between the observations was first tested. The results of the autocorrelation test showed a Durbin-Watson value of 1.980, which is relatively close to 2, suggesting that the observations are independent of each other. The results of the item multicollinearity test showed that the VIF were <10 and 1/VIF were >0.1, suggesting that there was no multicollinearity problem. Pearson correlation coefficients between the entry scores and the total questionnaire scores were then tested (Table 3). The results of Pearson correlation coefficients showed that the entry scores were significantly correlated with the total questionnaire scores (*p*-value < 0 01), and all Pearson correlation coefficients were >0.40. Exploratory factor analysis (inclusion criteria were common factor loadings ≧0.4) was then conducted for all items based on theoretical concepts (29) (Table 4). The results of the exploratory factor analysis showed that the eigenvalues of the four common factors were 12.257, 1.896, 1.530, and 1.115, respectively, with a cumulative variance explained of 69.987%. The final assessment questionnaire obtained was 4 dimensions (interest support, material support, emotional support, and behavioral support) with 24 items (Table 5).

**Table 3:** Pearson correlation coefficient table.

**Coding**	**Pearson correlation coefficient**	**Coding**	**Pearson correlation coefficient**	**Coding**	**Pearson correlation coefficient**
Q1	0.584**	Q15	0.687**	Q29	0.780**
Q2	0.611**	Q16	0.688**	Q30	0.794**
Q3	0.699**	Q17	0.734**	Q31	0.770**
Q4	0.654**	Q18	0.741**	Q32	0.811**
Q5	0.642**	Q19	0.713**	Q33	0.718**
Q6	0.681**	Q20	0.722**	Q34	0.736**
Q7	0.671**	Q21	0.759**	Q35	0.764**
Q8	0.694**	Q22	0.755**	Q36	0.766**
Q9	0.621**	Q23	0.798**	Q37	0.740**
Q10	0.615**	Q24	0.774**	Q38	0.789**
Q11	0.760**	Q25	0.702**	Q39	0.789**
Q12	0.730**	Q26	0.669**	Q40	0.776**
Q13	0.657**	Q27	0.783**	Q41	0.786**
Q14	0.691**	Q28	0.750**	Q42	0.750**

^**^P < 0.01.

**Table 4:** Standardized factor loading tables.

**Coding**	**Factor loading**				**Common factor variance**
	**Factor 1**	**Factor 2**	**Factor 3**	**Factor 4**	
Q1		0.714			0.610
Q2		0.798			0.729
Q3		0.751			0.760
Q4		0.731			0.685
Q5		0.782			0.749
Q6		0.707			0.694
Q7		0.585			0.648
Q9			0.656		0.612
Q10			0.743		0.668
Q11			0.579		0.676
Q12			0.619		0.658
Q13			0.770		0.733
Q14			0.700		0.672
Q18	0.609				0.610
Q20	0.751				0.702
Q21	0.685				0.689
Q22	0.742				0.739
Q24	0.757				0.759
Q25	0.797				0.732
Q26	0.752				0.661
Q30				0.685	0.751
Q31				0.795	0.835
Q33				0.809	0.790
Q37				0.622	0.632

**Table 5:** Dimensions and items of PEPSQ.

**Dimension**	**Item description**
**Interest support**
Q1	I have friends who have the same exercise interests as me
Q2	I have friends who share my exercise purpose
Q3	I have friends who like the same sports stars as me
Q4	I have friends with whom I share the same sports views and ideas
Q5	I have friends with whom I get along well in sports
Q6	I have friends who like the same sports brands as me
Q7	I have friends with whom I talk about solving exercise problems
**Material support**
Q9	My friend provided me with books for physical exercise
Q10	My friend provided me with some places to exercise
Q11	My friend provided me with some water or drinks for physical exercise
Q12	My friend provided me with some supplementary food for physical exercise
Q13	My friend helped me buy some clothes for physical exercise (e.g., sports clothes, sports shoes, etc.)
Q14	My friend bought me some sports equipment (e.g., basketball, badminton racket/ball, etc.)
**Emotional support**
Q18	When I want to quit sticking to my exercise program, my friends encourage me to keep going
Q20	My friend will comfort me when I have difficulties in physical exercise
Q21	My friend will work with me to solve problems I encounter in physical exercise
Q22	My friend understands how I feel in physical exercise
Q24	My friend encourages me when I am unable to accomplish my exercise goals
Q25	My friends encourage me when I feel inferior because of my poor athletic skills
Q26	My friends take care of me when I get injured in sports
**Behavioral support**
Q30	When I don't want to play sports, my friend invites me to play sports
Q31	Even if my friends don't play sports, they will be there for me when I play sports
Q33	Even if my friends have other things to do, they often make time to exercise with me
Q37	My friends will watch some sports programs with me

**Figure 2:**
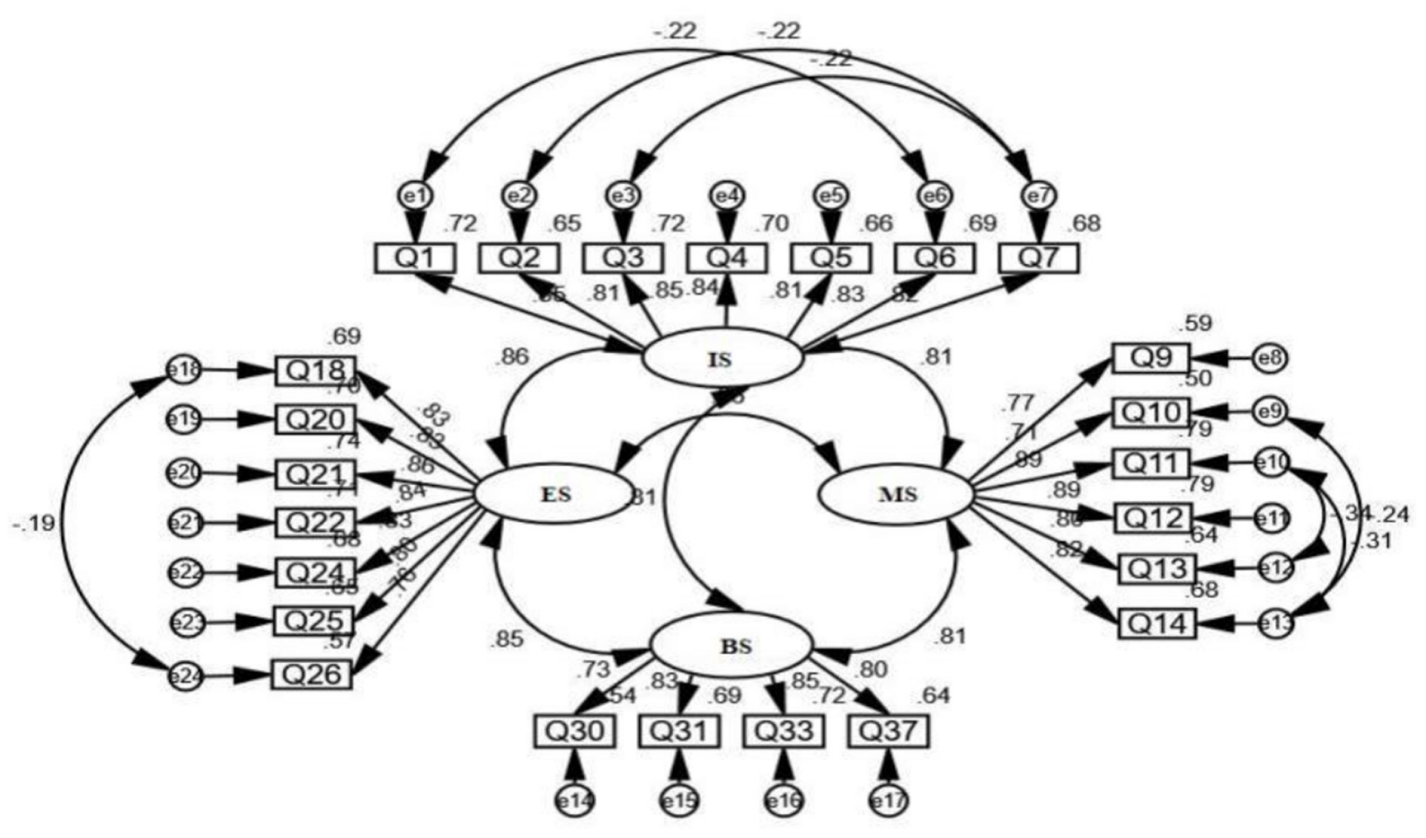
Estimated results of standardized path coefficients of final model after revision. IS, Interest support; ES, Emotional support; BS, Behavioral support; MS, Material support.

This study used data from 1,219 university students for questionnaire analysis to verify the independence of the questionnaire dimensions. The independence between the observations was first tested. The results of the autocorrelation test showed a Durbin-Watson value of 2.069, which exceeds 2, indicating that the observations are independent of each other. The results of the item multicollinearity test showed that VIF <10 and 1/VIF >0.1, indicating that there is no multicollinearity problem.After testing for entry independence, a validation factor analysis of the questionnaire was conducted using Amos 23.0 software. In the initial model (Table 6), although RMSEA = 0.080 and X^2^/df = 4.28 for the model, the significance probability value of *p* < 0.05 reached a significant level, indicating that the fitness of the hypothetical model plot to the observed data needs to be improved and the model needs to be further revised. Therefore, referring to Wu's suggestion (30), it is assumed that for the model to achieve a better fit, a better approach to model revision is to release certain assumptions. The initial model assumes that there is no correlation between the error variables and then, according to the AMOS correction indicator prompt, it is possible to find some degree of covariation in the error variables of some observed variables. If they are reset to have a covariate relationship with each other, the fitness of the model can be optimized. Thus, this study corrected the model according to the maximum correction value class, releasing multiple assumptions one at a time. The revised model obtained after multiple releases had *X*^2^/df = 3.41 and RMSEA = 0.074, with a significance probability value of *p* > 0.05, which did not reach a significant level, indicating a better fit of the hypothesis model plot to the observed data (see Figure 2). Factor loadings for all entries were above 0.7, indicating good convergent validity for each factor. The revised RMSEA was within an acceptable fit range, although it did not reach the best value recommended by Hu and Bentler (31).

**Table 6:** The results of the questionnaire's structural validity test.

**Model**	**X^2^/df**	**TLI**	**CFI**	**RMSEA**
Initial model	4.28	0.891	0.901	0.080
Revised model	3.41	0.905	0.917	0.074

### Reliability analysis of the questionnaire

In this study, the reliability of PEPSQ was tested by homogeneity test (Cronbach's alpha coefficient) and split-half coefficient (Spearmen-Brown correlation coefficient). The specific test results are shown in Table 7. The results of the homogeneity test and the split-half coefficient test indicate that the reliability of the PEPSQ is good. Meanwhile, the results of the correlation analysis between the total score of each dimension of PEPSQ and the total score of the questionnaire showed that the correlation coefficients of interest support, material support, emotional support, and behavioral support and the total score of the questionnaire were 0.773, 0.868, 0.884, and 0.914, respectively, indicating that PEPSQ has good reliability (32).

**Table 7:** Questionnaire dimensions and reliability coefficients.

**Dimension**	**Number**	**Cronbach's**	**Spearman-brown**
	**of items**	**α**	**correlation**
			**coefficient**
Interest support	7	0.900	0.851
Material support	6	0.890	0.856
Emotional support	7	0.913	0.876
Behavioral support	4	0.844	0.846
PEPSQ	24	0.902	0.899

### Evidence of generalization

The overall Cronbach's α coefficient of the PEPSQ was 0.902, indicating that the PEPSQ has good internal consistency. Table 8 shows the results of the analysis of entry reliability, and the Cronbach's α coefficient and corrected total correlation (CITC value) after removing an entry are provided in the table, respectively.

**Table 8:** Results of the reliability analysis of the items.

**Coding**	**CITC**	**The alpha coefficient**	**Cronbach alpha**
		**of the deleted term**	**coefficient**
Q1	0.625	0.901	0.902
Q2	0.492	0.901	
Q3	0.520	0.901	
Q4	0.539	0.901	
Q5	0.495	0.901	
Q6	0.626	0.901	
Q7	0.617	0.901	
Q9	0.640	0.900	
Q10	0.571	0.901	
Q11	0.766	0.894	
Q12	0.674	0.900	
Q13	0.703	0.900	
Q14	0.692	0.900	
Q18	0.830	0.892	
Q20	0.724	0.900	
Q21	0.665	0.901	
Q22	0.697	0.901	
Q24	0.678	0.901	
Q25	0.660	0.901	
Q26	0.711	0.900	
Q30	0.727	0.900	
Q31	0.751	0.900	
Q33	0.784	0.900	
Q37	0.680	0.901	

This study was conducted in both test samples and the sampling strategy was tested for adequacy in establishing a reliable hypothesis for identifying the level of perceived peer support among college students in physical exercise setting. Using data from a sample of 1,219 college students, the study ranked the PEPSQ scores from highest to lowest. The respondents in the top and bottom 25% of the total PEPSQ scores were named as high and low subgroups, and independent sample *t*-tests were conducted for each entry. The results of the analysis are presented in Table 9. The results indicate that the t-statistic (i.e., the decision value) for each entry was >10 and that the scores were significant between the high and low subgroups (*p*-value < 0.01). The findings suggest that the sampling strategy of this study is sufficient to establish a reliable identification of college students' perceived level of peer support in physical exercise settings.

**Table 9:** Results of the discrimination analysis of items.

**Coding**	**Mean** ±**SD**		***T*-value**	***P*-value**
	**High grouping**	**Low grouping**		
	**(*N* = 318)**	**(*N* = 304)**		
Q1	3.98 ± 0.86	2.56 ± 0.93	10.593	<0.01
Q2	4.09 ± 0.71	2.76 ± 1.01	10.196	<0.01
Q3	4.14 ± 0.64	2.47 ± 0.90	14.383	<0.01
Q4	4.07 ± 0.77	2.60 ± 0.95	11.310	<0.01
Q5	4.18 ± 0.66	2.86 ± 0.94	10.884	<0.01
Q6	4.23 ± 0.66	2.72 ± 0.96	12.251	<0.01
Q7	4.09 ± 0.71	2.58 ± 1.01	11.529	<0.01
Q9	3.89 ± 0.79	2.48 ± 0.96	10.769	<0.01
Q10	3.86 ± 0.91	2.23 ± 0.88	12.223	<0.01
Q11	4.20 ± 0.68	2.22 ± 0.90	16.536	<0.01
Q12	4.00 ± 0.73	2.26 ± 0.86	14.579	<0.01
Q13	3.73 ± 0.89	2.06 ± 0.85	12.875	<0.01
Q14	4.02 ± 0.68	2.27 ± 0.91	14.603	<0.01
Q18	4.10 ± 0.61	2.40 ± 0.99	13.795	<0.01
Q20	4.12 ± 0.66	2.57 ± 0.91	13.069	<0.01
Q21	4.16 ± 0.58	2.52 ± 0.90	14.506	<0.01
Q22	4.26 ± 0.55	2.66 ± 0.90	14.369	<0.01
Q24	4.27 ± 0.59	2.60 ± 0.86	15.024	<0.01
Q25	4.21 ± 0.64	2.75 ± 0.87	12.719	<0.01
Q26	4.27 ± 0.56	2.83 ± 1.03	11.605	<0.01
Q30	4.16 ± 0.73	2.32 ± 0.89	15.152	<0.01
Q31	4.04 ± 0.77	2.15 ± 0.95	14.644	<0.01
Q33	3.92 ± 0.82	2.18 ± 0.92	13.453	<0.01
Q37	4.14 ± 0.72	2.30 ± 0.94	14.778	<0.01

### Evidence of extrapolation

The Exercise Social Support Scale was used as the validity standard of PEPSQ. The correlation test results showed that the correlation coefficients of the PEPSQ dimension scores and total scores with the exercise social support scale dimension scores and total scores reached a significant level of *P* < 0.01. This indicates that the PEPSQ has good validity of the validity scale correlation validity to identify the perceived peer support of college students in the physical exercise environment. The results of the analysis are shown in Table 10.

**Table 10:** Correlation analysis results.

	**Interest support**	**Material support**	**Emotional support**	**Behavioral support**	**PEPSQ**
Instrumental support	0.453**	0.630**	0.514**	0.678**	0.659**
Informational support	0.479**	0.547**	0.490**	0.543**	0.603**
Affective support	0.468**	0.457**	0.648**	0.464**	0.608**
Peer support	0.419**	0.622**	0.554**	0.627**	0.647**
ESSS	0.517**	0.651**	0.624**	0.669**	0.720**

^**^P < 0.01.

This study further examined the retest reliability of the PEPSQ at 2-week intervals. The results showed that the data of both tests reached a significance level of *p* < 0.01 for all dimensions, and the retest reliability was above 0.7 for all dimensions, indicating that the PEPSQ measures have some stability.

### Evidence of implications

The results of this study suggest that researchers or educators can use the PESCQ to differentiate between groups of college students who participate in physical exercise or who do not, and target interventions to different groups, which has implications for practical application. The results of the analysis are shown in Table 11.

**Table 11:** Comparison of PEPSQ scores of different groups.

	**Regular exercise**	**No regular**	***T*-value**
	**group**	**exercise group**	
	**(*n* = 155)**	**(*n* = 1,064)**	
–	24.38 ± 5.18	21.31 ± 5.05	4.727**
Material support	19.22 ± 4.68	16.61 ± 4.49	4.448**
Emotional support	24.56 ± 5.18	21.90 ± 5.39	4.044**
Behavioral support	12.89 ± 3.41	11.22 ± 3.44	3.807**
PEPSQ total score	81.05 ± 15.81	71.04 ± 15.42	5.035**

^**^P < 0.01.

## Discussion

With reference to Kane's validity framework, this study presents the research process and results of this study by inference category. As noted above, this drove the data collection and analysis plan for this study. This study operationalised the corresponding validity evidence that the study needed to demonstrate in response to Kane's conceptual definition of scoring, generalization, extrapolation, and implications. This evidence can help to develop support for the validity of the PEPSQ, as well as inferences based on the scores generated.Kane's validity framework emphasizes a chain of inferences from score generation to inference about the test taker's decision, a chain that can be operationalised as a path that must be followed before sufficient evidence can be obtained. Therefore, the operationalisation of this step is used as a guide to report the results of this study.

To form validity arguments, Kane suggested evaluating the evidence and deciding whether to accept or reject it, and/or modify the process and/or the proposed use. In the scoring evidence, evidence of PEPSQ dimensional independence, and evidence of entry differentiation were validated. In the Generalization evidence, both sampling data showed that the sampling strategy of this study was sufficient to establish a reliable identification of college students' perceived level of peer support in physical exercise settings. In the Extrapolation evidence, the results of the correlation analysis using the Exercise Social Support Scale with this questionnaire showed that the assessment process of this study would predict future real-world performance in real-world physical exercise settings. Also the small-sample retest reliability in meeting the hypothesis (medium to high level) indicates that the PEPSQ measures have some stability. In Implications evidence, given our homogeneity and highly selected participants, the study tested the assessment results to predict physical exercise behavior in real exercise settings. The results of the study showed that the regular exercise group had significantly higher scores and total scores in interest support, material support, emotional support and behavioral support than the university students in the no regular exercise group. It is suggested that the assessment results of this study can predict the real behaviors in physical exercise settings.

Although the four inferred results of this study are relatively positive to illustrate the validity of the PEPSQ. However, this series of processes is primarily intended to illustrate that this study's argument for PEPSQ validity is not a conclusion, but rather represents a series of positive steps in research aimed at building and refining the evidence for PEPSQ validity.

Applying Kane's validity framework, this study's argument for the validity of the PEPSQ is demonstrated through an operationalised argument for four processes: scoring, generalization, extrapolation, and implications. Reflecting on the entire process of this study, the Kane framework helped structure the study's organizational and analytical framework. The validity of the PEPSQ is a chain of evidence strung together. However, in this study, challenges were encountered in deciding how to prioritize the collection and reporting of evidence across the four inferential dimensions. Because there is a paucity of research literature related to physical exercise peer support, this made it difficult for the research team to determine from the available studies which weak and problematic links must be prioritized in the design of this study. Therefore, the research design for the weak and problematic links in this study may not be adequate and may leave important evidence gaps in the validity argument.

At the same time, there are some limitations in this study. First, the stability of the study results may be affected by the sample data in this study due to the sampling method, and further validation through a large national sample data is needed in the future. Second, these data were obtained from the subjects' self-assessment reports, and the data results may be affected by the subjects' text reading comprehension ability, and their understanding of the questionnaire items may vary. Finally, the evidence for the four inferential processes in this study was based only on subjects who completed the questionnaire in its entirety, which resulted in a lower error rate for the questionnaire, but this may have partially influenced the results of the test.

## Conclusion

The PEPSQ, developed in this study, has four dimensions and twenty-four items. This study used the Kane validity framework to identify and examine the validity process of the PEPSQ. Evidence based on the four inferential processes of scoring, induction, extrapolation, and influence of the Kane framework supports that the PEPSQ can be used to measure the level of perceived peer support in physical exercise settings among Chinese college students.

## Data availability statement

The raw data supporting the conclusions of this article will be made available by the authors, without unduereservation.

## Ethics statement

The studies involving human participants were reviewed and approved by the Ethics Review and Approval of the Academic Committee of the Physical Education College of Guizhou Normal University (No. 20210310). The patients/participants provided their written informed consent to participate in this study.

## Author contributions

XY, LL, and NS conceived the study and performed data analysis and interpretation. LL and XY prepared the manuscript. LZho, LZha, YY, and JY participated in data collection. XZ was involved in the revision of the paper. All authors have read and approved the final manuscript.

## Funding

This study was funded by the Doctoral Fund of Guizhou Normal University [No. GZNUD (2018)-8], the Youth Growth Project Fund of Guizhou Provincial Department of Education [Qianjiaohe KY (2021) 291], and the Guizhou Provincial Education Planning Fund Project (2021A058).

## Conflict of interest

The authors declare that the research was conducted in the absence of any commercial or financial relationships that could be construed as a potential conflict of interest.

## Publisher's note

All claims expressed in this article are solely those of the authors and do not necessarily represent those of their affiliated organizations, or those of the publisher, the editors and the reviewers. Any product that may be evaluated in this article, or claim that may be made by its manufacturer, is not guaranteed or endorsed by the publisher.

## References

[1] Guo RP Xu JF Li L Lu YL Feng LS. A comparative study of Chinese and foreign adolescent physical health assessment systems. Chin J Sports Sci Technol. (2019) 55:3–13. 10.16470/j.csst.2019091

[2] Research Group on Physical Fitness and Health of Chinese Students. Survey Report on Physical Fitness and Health of Chinese Students. (2014). Beijing: Higher Education Press (2018).

[3] Guo JJ Yang H. China Youth Sports Development Report. Beijing: Social Sciences Literature Publishing House (2015).

[4] Xu Y Chen JF Xu Y Liao WH. Research on the current situation and countermeasures of the management of physical health monitoring and evaluation of college students in Guangdong Province. Higher Educ Expl. (2020) 8:106–10. Available online at: https://www.cnki.com.cn/Article/CJFDTotal-GJTA202008018.htm

[5] Gang J. Exploration on the practice of physical health management of contemporary college students. Chin School Health J. (2021) 42:1121. Available online at: https://www.cnki.com.cn/Article/CJFDTotal-XIWS202107042.htm33686439

[6] Prochaska JJ Rodgers MW Sallis JF. Association of parent and peer support with adolescent physical activity. Res Quart Exerc Sport. (2002) 73:206–10. 10.1080/02701367.2002.10609010 12092896

[7] Mead S Hilton D Curtis L. Peer support: a theoretical perspective. Psychiatr Rehabil J. (2001) 25:134–41. 10.1037/h0095032 11769979

[8] Wentzel KR Battle A Russell SL Looney LB. Social supports from teachers and peers as predictors of academic and social motivation. Cont Educat Psychol. (2010) 35:193–202. 10.1016/j.cedpsych.2010.03.002

[9] Fitzgerald A Fitzgerald N Aherne C. Do peers matter? A review of peer and/or friends' influence on physical activity among American adolescents. J Adol. (2012) 35:941–58. 10.1016/j.adolescence.2012.01.002 22285398

[10] Chen H Sun H Dai J. Peer support and adolescents' physical activity: The mediating roles of self-efficacy and enjoyment. J Pediatric Psychol. (2017) 42:569–77. 10.1093/jpepsy/jsw103 28158660

[11] Reimers AK Schmidt SCE Demetriou Y Marzi I Woll A. Parental and peer support and modelling in relation to domain-specific physical activity participation in boys and girls from Germany. PLoS ONE. (2019) 14:e0223928. 10.1371/journal.pone.0223928 31665192 PMC6821055

[12] Sylvia-Bobiak S Caldwell LL. Factors related to physically active leisure among college students. Leisure Sci. (2006) 28:73–89. 10.1080/0149040050033272823800133

[13] Zimet GD Dahlem NW Zimet SG Farley GK. The multidimensional scale of perceived social support. J Person Assess. (1988) 52:30–41. 10.1207/s15327752jpa5201_2

[14] Mostafaei Alaei M Hosseinnezhad H. The development and validation of peer support questionnaire (PSQ). J Teach Lang Skills. (2020) 39:67–109. 10.22099/jtls.2021.38853.2906

[15] Kerres Malecki C Kilpatrick Demary M. Measuring perceived social support: development of the child and adolescent social support scale (CASSS). Psychol School. (2002) 39:1–18. 10.1002/pits.10004

[16] Barrera M Sandler IN Ramsay TB. Preliminary development of a scale of social support: studies on college students. Am J Commun Psychol. (1981) 9:435–47. 10.1007/BF0091817429033116

[17] Chen NY. Research on the Relationship Between Internet Addiction, Peer Support and Overall Self-Worth of College Students. Hangzhou: Zhejiang University (2007).

[18] Helgeson V Gottlieb BH. Support Groups. Social Support Measurement and Intervention: A Guide for Health and Social Scientists. Oxford: Oxford University Press (2000).

[19] Wills TA Shinar O. Measuring Perceived and Received Social Support. Social Support Measurement and Intervention: A Guide for Health and Social Scientists. Oxford University Press (2000). p. 86–135. 10.1093/med:psych/9780195126709.003.0004

[20] Viswesvaran C Sanchez JI Fisher J. The role of social support in the process of work stress: a meta-analysis. J Voc Behav. (1999) 54:314–34. 10.1006/jvbe.1998.16619325800

[21] Rebeiro Gruhl KL LaCarte S Calixte S. Authentic peer support work: challenges and opportunities for an evolving occupation. J Mental Health. (2016) 25:78–86. 10.3109/09638237.2015.1057322 26397981

[22] Zhong T Hu L Liu XG. Establishment and reliability and validity of exercise social support scale. Sports Sci Res. (2019) 40:42–7. Available online at: http://www.cqvip.com/qk/82694x/201906/7100387899.html

[23] Sallis JF Grossman RM Pinski RB Patterson TL Nader PR. The development of scales to measure social support for diet and exercise behaviors. Prev Med. (1987) 16:825–36. 10.1016/0091-7435(87)90022-3 3432232

[24] Farias Júnior JC Mendonça G Florindo AA Barros MVGD. Reliability and validity of a physical activity social support assessment scale in adolescents-ASAFA Scale. Rev Brasil Epidemiol. (2014) 17:355–70. 10.1590/1809-4503201400020006ENG 24918409

[25] Cook DA Brydges R Ginsburg S Hatala R. A contemporary approach to validity arguments: a practical guide to Kane's framework. Med Educ. (2015) 49:560–75. 10.1111/medu.12678 25989405

[26] Kane MT. Validating the interpretations and uses of test scores. J Educ Meas. (2013) 50:1–73. 10.1111/jedm.1200031205462

[27] Anderssen N Wald B. Parental and peer influences on leisure-time physical activity in young adolescents. Res Quart Exerc Sport. (1992) 63:341–8. 10.1080/02701367.1992.10608754 1439157

[28] Hohepa M Scragg R Schofield G Kolt GS Schaaf D. Social support for youth physical activity: importance of siblings, parents, friends and school support across a segmented school day. Int J Behav Nutr Phys Act. (2007) 4:1–9. 10.1186/1479-5868-4-54 17996070 PMC2186357

[29] Mao FZ Han YF Fang Y. Research progress of statistical screening methods for scale entries. Mod Prev Med. (2015) 42:1–3. Available online at: https://core.ac.uk/download/pdf/41438069.pdf

[30] Wu ML. Structural Equation Modeling - Operations and Applications of AMOS. 2nd ed. Chongqing: Chongqing University Press (2010).

[31] Hu LT Bentler PM. Cutoff criteria for fit indexes in covariance structure analysis: conventional criteria versus new alternatives. Struct Equ Modeling. (1999) 6:1–55. 10.1080/10705519909540118

[32] Nunnally JC Bernstein IH. Factor analysis I: The general model and variance condensation. In: Psychometric Theory. 3rd ed. New York, NY: McGraw Hill (1994). p. 447–90.

