# Supplementary material for: Evaluation of the validity of the physical exercise peer support questionnaire for college students
Source: Front Public Health. 2022 Aug 1;10:871306. doi: 10.3389/fpubh.2022.871306 (PMC9390216; doi:10.3389/fpubh.2022.871306)
Supplement: Supplementary file 1 [file Data_Sheet_1.PDF]

IRB 审核编码（IRB NO.）：20210310　　申请日期（Date of Application）：2021 年 3 月 10 日

贵州师范大学体育学院研究项目伦理审核申请表
（RESEARCH PROJECT ETHICAL REVIEW APPLICATION FORM, PHYSICAL EDUCATION INSITUTE, GUIZHOU NORMAL UNIVERSITY）

基本信息（GENERAL INFORMATION）

1. 课题题目（Project Title）：大学生身体素养测评研究（A study on physical literacy assessment of university students）

2. 课题简介(Brief Summary): 请在此用 200-300 字对申请课题进行简要描述。此简介将作为对该项目的介绍被委员会存档。

身体素养（Physical literacy，PL）是指“重视和积极参与身体活动的动机、信心、身体能力、知识和理解[1]”，是个体实现健康生活方式的能力，对个体的身心健康发展十分重要。身体素养水平较高的个体将更加有自信和能力参与各种身体活动，而身体素养水平较低的个体其身体活动行为也更少。将身体素养作为促进个体身体活动的重要手段，需要借助身体素养的测评工具，去帮助了解人们的身体素养水平。据我们对已发表文章的了解，目前可用于大学生身体素养测评的工具较少，因此，为了满足中国大学生大规模身体素养测评的需求，我们旨在设计一个初步问卷来确定大学生的身体素养。

注：是否有与这项新课题类似或相关的前序课题已通过研究所伦理审核？是□否☑

3. 研究人员（Project Personnel）：请分别列出本课题的研究负责人以及研究参加人员。研究参加人员包括所有与实验被试或者被试数据有直接接触的人员。

3a. 研究负责人（Principal Investigator）

姓名（Name）：罗琳（Luo Lin）	单位（Institute）：贵州师范大学体育学院（School of Physical Education, Guizhou Normal University)
电话（Tel）：13765070212	手机（Cell）：13765070212
E-mail：5925860@qq.com	

3b. 研究参加人员（Participating Researchers）：

姓名（Name）：	单位（Institute）
1.宋乃庆（Song Naiqing）	西南大学基础教育研究中心
2.杨豪(Yang Hao)	华东师范大学附属中旭学校
3.黄家洪(Huang Jiahong)	贵州师范大学体育学院
4.周玲（Zhou Ling）	贵州师范大学体育学院
5.张利平(Zhang Liping)	贵州师范大学体育学院
6.	
7.	
8.	
9.	
10.	
11.	
12.	

4. 经费来源（Funding Source）：

本研究的经费来自华东师范大学-中旭博士后工作站基金（No.2019001）、贵州省教育厅青少年成长工程基金（黔教他 KY[2021]291）资助，以及贵州省教育规划基金项目（2021A058）。

审核豁免（Review Exemptions）

部分有人类被试参与的研究可以申请审核豁免，这可以减少申请和审核的要求，加快审核的速度。请求审核豁免的研究者请在以下空白陈述豁免理由。【注：仅针对二手数据（如学校系统中的学生成绩、校医院的学生健康数据等）】

研究课题介绍（Research Project Introduction）

1. 研究目标（Specific Aims）：（限 300 字）

通过收集该问卷的有效性证据来衡量中国大学生体育素养自我评估问卷的有效性。

2. 研究背景和研究意义（Background and Significance）：（限 600 字）

体育素养（PL）是促进个体体育活动的重要工具，个体的体育素养水平会影响其体育活动行为。目前，中国大学生的体质是一个突出的问题，评估大学生的体育素养可以为今后进一步促进大学生体质和精确干预大学生的体育活动行为提供工具和方向。本研究旨在开发一份大学生体育素养问卷（CSPLQ），以解决目前中国大学生缺乏体育素养评估工具的问题。

3. 研究被试（Research Subjects）： 请提供研究被试的人数、年龄阶段，并注明本研究是否旨在招募有特殊需要的人群（例如，弱势群体，病人，不能独立作决定的个体，儿童，孕妇等）。

大专、本科、研究生

4. 研究设计、方法与程序（Research Design, Method, and Procedure）

本项研究的研究程序共包含四个步骤：条目的生成、参与者招募、有效性过程和评分。其中，采用 AERA-APA (1999) 推荐的有效性框架，使用内容、响应过程、内部结构、与其他变量的相关性和结果来衡量有效性过程。整个研究过程会进行 3 次问卷测试。

研究伦理审核（Research Ethics Review）

1. 风险与最小化风险措施（Risks and Measures to Minimize Risks）：请根据申请课题的实际情况在每一项风险后面对可能存在风险的几率进行描述：非常常见（发生率> 50%）；常见（发生率> 25%；有可能（发生率介于 10-25%）；不常见（发生率介于 1-10%）；罕见（发生率< 1%）。

a. 心理风险（Psychological Risks）	
– 痛苦的情绪	不常见
– 尴尬	不常见
– 由于影响隐私而引起的情绪波动	罕见
– 其他	罕见
b. 社会风险（Social Risks）	
– 在社交圈中名誉或者地位受损	罕见
– 对研究被试所代表的更大群体带来伤害（例如，歧视）	罕见
– 其他	罕见
c. 经济风险（Economic Risks）	
– 收入的损失	罕见
– 失业或失去社会保障	罕见
– 职场名誉或地位的受损	罕见
– 其他	罕见
d. 法律风险（Legal Risks）	
– 公开非法活动	罕见
– 公开渎职	罕见

– 其他	罕见
e. 生理风险（Physical Risks）	
– 生理疼痛	罕见
– 身体不舒适	罕见
– 身体伤害	罕见
– 其他	罕见

2. 受益（Benefits）：请描述被试参与此研究课题将会得到的直接与间接的受益。

无

3. 被试隐私保护（Participants Privacy Protection）

本研究的结果可能会在学术期刊/书籍上，或者用于教学。除非得到被试的允许，被试的名字或者其他可以确认被试的信息将不会在任何发表或教学材料中出现。另外，在本研究过程中取得的能够确定被试身份的照片、录音或录像，都将在得到被试的书面允许后才会使用。

签名（Signature）：　　**日期（Date）**

4. 数据保密（Confidentiality of the Data）

本研究所产生的所有数据将被储存在有安全保障的研究室中，绝不在数据采集、分析、发布的过程中泄露任何有关被试的个人信息。

签名（Signature）：　　**日期（Date）：**

5. 被试知情声明（Participants Consent Statement）

本研究将告知被试本研究的目的、过程、可能的风险和副作用以及潜在的收获和费用，并最大可能地回答被试提出的与研究有关的问题。

签名（Signature）：　　**日期（Date）：**

6. 研究负责人保证书（Certification of Principal Investigator）：

本人声明所填写内容属实，并将严格按照申请书中有关内容从事实验和研究。本人并表示严格遵守国家法律、贵州师范大学体育学院有关规定，同时保护被试的健康、权益和隐私。本人有责任将实验中出现的问题如实向体育学院汇报，并按照研究所的要求改正。

签名（Signature）：　　**日期（Date）**

审批意见及结果

1. 学术委员会意见及保证书（Opinions and Certification of the Scientific Review Committee）：

IRB 审核编码（IRB NO.）：20210310　　　申请日期（Date of Application）：2021 年 3 月 10 日

对该研究申请人主持此项工作的意见（Opinion of the competency of the investigator(s) to conduct this project）：　同意☑　　不同意 □

学术委员会评审专家签名　　　　日期

（Signature of Experts of the Scientific Review Committee）（Date）　2021.3.10

以下签名确认本委员会已经考察了研究申请人的科研水平和所提科研项目的科研价值，并同意该研究申请人主持此项研究工作。

贵州师范大学体育学院

学术委员会主席签名　　　日期 2021.3.10

（Signature of Chair of Scientific Review Committee）（Date）
