# Supplementary material for: Evaluation of the validity of the physical exercise peer support questionnaire for college students
Source: Front Public Health. 2022 Aug 1;10:871306. doi: 10.3389/fpubh.2022.871306 (PMC9390216; doi:10.3389/fpubh.2022.871306)
Supplement: Supplementary file 2 [file Data_Sheet_2.docx]

**Supplementary material 1：**

Pre-Test Questionnaire of PEPSQ

Please read each item carefully to understand the meaning, and then according to your actual feelings in the last week, choose the answer that suits you best and tick the corresponding number with "√".

(1. Completely disagree 2. Mostly disagree 3. General agreement 4. Mostly agree 5. Completely agree)

| Coding | Item description |  |  |  |  |  |
| --- | --- | --- | --- | --- | --- | --- |
| Q1 | I have friends who have the same exercise interests as me | 1□ | 2□ | 3□ | 4□ | 5□ |
| Q2 | I have friends who share my exercise purpose | 1□ | 2□ | 3□ | 4□ | 5□ |
| Q3 | I have friends who like the same sports stars as me | 1□ | 2□ | 3□ | 4□ | 5□ |
| Q4 | I have friends with whom I share the same sports views and ideas | 1□ | 2□ | 3□ | 4□ | 5□ |
| Q5 | I have friends with whom I get along well in sports | 1□ | 2□ | 3□ | 4□ | 5□ |
| Q6 | I have friends who like the same sports brands as me | 1□ | 2□ | 3□ | 4□ | 5□ |
| Q7 | I have friends with whom I talk about solving exercise problems | 1□ | 2□ | 3□ | 4□ | 5□ |
| Q8 | I have friends who follow similar sports information to me | 1□ | 2□ | 3□ | 4□ | 5□ |
| Q9 | My friend provided me with books for physical exercise | 1□ | 2□ | 3□ | 4□ | 5□ |
| Q10 | My friend provided me with some places to exercise | 1□ | 2□ | 3□ | 4□ | 5□ |
| Q11 | My friend provided me with some water or drinks for physical exercise | 1□ | 2□ | 3□ | 4□ | 5□ |
| Q12 | My friend provided me with some supplementary food for physical exercise | 1□ | 2□ | 3□ | 4□ | 5□ |
| Q13 | My friend helped me buy some clothes for physical exercise (e.g.sports clothes, sports shoes, etc.) | 1□ | 2□ | 3□ | 4□ | 5□ |
| Q14 | My friend bought me some sports equipment (e.g. basketball, badminton racket/ball, etc.) | 1□ | 2□ | 3□ | 4□ | 5□ |
| Q15 | My friends reward me when I reach my workout goals | 1□ | 2□ | 3□ | 4□ | 5□ |
| Q16 | My friends use rewards to motivate me to exercise | 1□ | 2□ | 3□ | 4□ | 5□ |
| Q17 | My friends often encourage me to exercise | 1□ | 2□ | 3□ | 4□ | 5□ |
| Q18 | When I want to quit sticking to my exercise program, my friends encourage me to keep going | 1□ | 2□ | 3□ | 4□ | 5□ |
| Q19 | My peers praise me when I accomplish my physical exercise goals | 1□ | 2□ | 3□ | 4□ | 5□ |
| Q20 | My friend will comfort me when I have difficulties in physical exercise | 1□ | 2□ | 3□ | 4□ | 5□ |
| Q21 | My friend will work with me to solve problems I encounter in physical exercise | 1□ | 2□ | 3□ | 4□ | 5□ |
| Q22 | My friend understands how I feel in physical exercise | 1□ | 2□ | 3□ | 4□ | 5□ |
| Q23 | My friends are willing to listen to my sports experience sharing | 1□ | 2□ | 3□ | 4□ | 5□ |
| Q24 | My friend encourages me when I am unable to accomplish my exercise goals | 1□ | 2□ | 3□ | 4□ | 5□ |
| Q25 | My friends encourage me when I feel inferior because of my poor athletic skills | 1□ | 2□ | 3□ | 4□ | 5□ |
| Q26 | My friends take care of me when I get injured in sports | 1□ | 2□ | 3□ | 4□ | 5□ |
| Q27 | My friends and I often do physical exercise together | 1□ | 2□ | 3□ | 4□ | 5□ |
| Q28 | My friends help me develop and implement my physical activity plan | 1□ | 2□ | 3□ | 4□ | 5□ |
| Q29 | My friends monitor my physical activity from time to time | 1□ | 2□ | 3□ | 4□ | 5□ |
| Q30 | When I don't want to play sports, my friend invites me to play sports | 1□ | 2□ | 3□ | 4□ | 5□ |
| Q31 | Even if my friends don't play sports, they will be there for me when I play sports | 1□ | 2□ | 3□ | 4□ | 5□ |
| Q32 | My friends help me unwind after my workout | 1□ | 2□ | 3□ | 4□ | 5□ |
| Q33 | Even if my friends have other things to do, they often make time to exercise with me | 1□ | 2□ | 3□ | 4□ | 5□ |
| Q34 | My friends help me when I have trouble exercising | 1□ | 2□ | 3□ | 4□ | 5□ |
| Q35 | My friends often remind me to exercise regularly | 1□ | 2□ | 3□ | 4□ | 5□ |
| Q36 | My friends tell me about the benefits of exercising regularly | 1□ | 2□ | 3□ | 4□ | 5□ |
| Q37 | My friends will watch some sports programs with me | 1□ | 2□ | 3□ | 4□ | 5□ |
| Q38 | My friend will give me some exercise advice and guidance (like corrective technique etc.) | 1□ | 2□ | 3□ | 4□ | 5□ |
| Q39 | My friends remind me of some exercise dos and don'ts | 1□ | 2□ | 3□ | 4□ | 5□ |
| Q40 | When I want to work out, friends give me information about venues and more | 1□ | 2□ | 3□ | 4□ | 5□ |
| Q41 | My friends often share their workout experiences with me | 1□ | 2□ | 3□ | 4□ | 5□ |
| Q42 | My friends and I often talk about sports related things | 1□ | 2□ | 3□ | 4□ | 5□ |

**Supplementary material 2：**

The Physical Exercise Peer Support Questionnaire（PEPSQ）

Please read each item carefully to understand the meaning, and then according to your actual feelings in the last week, choose the answer that suits you best and tick the corresponding number with "√".

(1. Completely disagree 2. Mostly disagree 3. General agreement 4. Mostly agree 5. Completely agree)

| Dimension | Item description |  |  |  |  |  |
| --- | --- | --- | --- | --- | --- | --- |
| Interest support | |  |  |  |  |  |
| 1 | I have friends who share my interests in exercising | 1□ | 2□ | 3□ | 4□ | 5□ |
| 2 | I have friends who exercise for the same purpose as me | 1□ | 2□ | 3□ | 4□ | 5□ |
| 3 | I have friends who share my sports preferences | 1□ | 2□ | 3□ | 4□ | 5□ |
| 4 | I have friends with whom I share a vision or idea of the movement | 1□ | 2□ | 3□ | 4□ | 5□ |
| 5 | I have friends with whom I get along well during physical exercise | 1□ | 2□ | 3□ | 4□ | 5□ |
| 6 | I have friends who encourage each other with me during physical exercise | 1□ | 2□ | 3□ | 4□ | 5□ |
| 7 | I have friends with whom I talk about or resolve difficulties with the physical exercise process | 1□ | 2□ | 3□ | 4□ | 5□ |
| Material support | |  |  |  |  |  |
| 8 | My friend offered me some physical exercise books | 1□ | 2□ | 3□ | 4□ | 5□ |
| 9 | My friend offered me some sports venues | 1□ | 2□ | 3□ | 4□ | 5□ |
| 10 | My friend provided me with some water or drinks for physical exercise | 1□ | 2□ | 3□ | 4□ | 5□ |
| 11 | My friend provided some supplementary food for physical exercise | 1□ | 2□ | 3□ | 4□ | 5□ |
| 12 | My friend helped me get some of the clothing I needed to exercise (like tracksuits, sneakers, etc.) | 1□ | 2□ | 3□ | 4□ | 5□ |
| 13 | My friend helped me get some sports equipment (eg basketball, badminton racket/ball, etc.) | 1□ | 2□ | 3□ | 4□ | 5□ |
| Emotional support | |  |  |  |  |  |
| 14 | When I can't keep exercising and want to give up, my friends will encourage me to keep going | 1□ | 2□ | 3□ | 4□ | 5□ |
| 15 | Friends comfort me when I'm having a hard time with my physical exercise | 1□ | 2□ | 3□ | 4□ | 5□ |
| 16 | My friends will share with me the problems encountered in physical exercise | 1□ | 2□ | 3□ | 4□ | 5□ |
| 17 | My friends understand how I feel during physical exercise | 1□ | 2□ | 3□ | 4□ | 5□ |
| 18 | Friends comfort me when I'm frustrated that I can't accomplish my physical exercise goals | 1□ | 2□ | 3□ | 4□ | 5□ |
| 19 | When I feel inferior because of my poor sports skills, my friends will encourage me | 1□ | 2□ | 3□ | 4□ | 5□ |
| 20 | When I get injured in sports, my friends will take care of me | 1□ | 2□ | 3□ | 4□ | 5□ |
| Behavioral support | |  |  |  |  |  |
| 21 | When I don't want to exercise, my friends invite me to exercise | 1□ | 2□ | 3□ | 4□ | 5□ |
| 22 | Even if my friends don't exercise, they will accompany me when I exercise | 1□ | 2□ | 3□ | 4□ | 5□ |
| 23 | Even when my friends have other business, they often plan to exercise with me | 1□ | 2□ | 3□ | 4□ | 5□ |
| 24 | My friends will watch some sports with me | 1□ | 2□ | 3□ | 4□ | 5□ |

**Supplementary material 2：**

The Exercise Social Support Scale（ESSS）

Please read each item carefully to understand the meaning, and then according to your actual feelings in the last week, choose the answer that suits you best and tick the corresponding number with "√".

(1. Completely disagree 2. Mostly disagree 3. General agreement 4. Mostly agree 5. Completely agree)

| Coding | Item description |  |  |  |  |  |
| --- | --- | --- | --- | --- | --- | --- |
| Q1 | Acknowledging my active participation in exercise | 1□ | 2□ | 3□ | 4□ | 5□ |
| Q2 | Reassure me when my physical activity creates negative emotions | 1□ | 2□ | 3□ | 4□ | 5□ |
| Q3 | Give a positive comment on the progress I have made with my physical training | 1□ | 2□ | 3□ | 4□ | 5□ |
| Q4 | Comfort me after my physical training injury | 1□ | 2□ | 3□ | 4□ | 5□ |
| Q5 | Affirmation of the fitness results I have achieved by exercising | 1□ | 2□ | 3□ | 4□ | 5□ |
| Q6 | I am sure I have mastered or improved my motor training skills | 1□ | 2□ | 3□ | 4□ | 5□ |
| Q7 | Yell me the opening hours of the exercise venue | 1□ | 2□ | 3□ | 4□ | 5□ |
| Q8 | Tell me about the precautions when exercising (such as safety matters, timely hydration, etc.) | 1□ | 2□ | 3□ | 4□ | 5□ |
| Q9 | Tell me about the charges for the exercise facility | 1□ | 2□ | 3□ | 4□ | 5□ |
| Q10 | Tell me how to get sports equipment or equipment | 1□ | 2□ | 3□ | 4□ | 5□ |
| Q11 | Provide me with exercise-related materials (such as videos, books, etc.) | 1□ | 2□ | 3□ | 4□ | 5□ |
| Q12 | Tell how to find a place to exercise | 1□ | 2□ | 3□ | 4□ | 5□ |
| Q13 | Help me set exercise goals | 1□ | 2□ | 3□ | 4□ | 5□ |
| Q14 | Arrange a place for me to exercise | 1□ | 2□ | 3□ | 4□ | 5□ |
| Q15 | Book a workout for me | 1□ | 2□ | 3□ | 4□ | 5□ |
| Q16 | Help me create an exercise plan | 1□ | 2□ | 3□ | 4□ | 5□ |
| Q17 | Monitor the achievement of my exercise goals | 1□ | 2□ | 3□ | 4□ | 5□ |
| Q18 | Provide me with equipment or equipment to exercise | 1□ | 2□ | 3□ | 4□ | 5□ |
| Q19 | Accompany me to exercise | 1□ | 2□ | 3□ | 4□ | 5□ |
| Q20 | Give me personal instruction on physical exercise techniques | 1□ | 2□ | 3□ | 4□ | 5□ |
| Q21 | Adjust your time to exercise with me | 1□ | 2□ | 3□ | 4□ | 5□ |
| Q22 | Provide assistance after my physical activity injury (eg dressing, buying medicine, etc.) | 1□ | 2□ | 3□ | 4□ | 5□ |
| Q23 | Introduce people who are active in exercising to work out with me | 1□ | 2□ | 3□ | 4□ | 5□ |
| Q24 | Massage me after exercise | 1□ | 2□ | 3□ | 4□ | 5□ |
